# A case report of secondary neurolymphomatosis showing selective nerve infiltration and massive lumbar plexus enlargement

**DOI:** 10.1186/s12883-021-02330-5

**Published:** 2021-07-27

**Authors:** Mai Hamaguchi, Norito Kokubun, Hadzki Matsuda, Hiroki Onuma, Reika Aoki, Wataru Takahashi, Kinuko Mitani, Keisuke Suzuki

**Affiliations:** 1 Department of Neurology, Dokkyo Medical University, 880 Kitakobayashi, Mibu, Shimotsuga 321-0293 Japan; 2 Department of Neurosurgery, Dokkyo Medical University, 880 Kitakobayashi, Mibu, Shimotsuga 321-0293 Japan; 3 Department of Hematology and Oncology, Dokkyo Medical University, 880 Kitakobayashi, Mibu, Shimotsuga 321-0293 Japan

**Keywords:** Cauda equina, Malignant lymphoma, Nerve biopsy, Neurolymphomatosis

## Abstract

**Background:**

Neurolymphomatosis (NL) is a rare manifestation of malignant lymphoma that shows selective infiltration to the peripheral nervous system primarily or secondarily. We report a patient with secondary NL caused by germinal center B-cell (GCB)-type diffuse large B-cell lymphoma (DLBCL) who showed selective infiltration of the lumbar plexus to the spinal cord and massive nerve enlargement resulting in severe pain.

**Case presentation:**

A 72-year-old female exhibited asymmetric motor and sensory impairments and pain in the lower limbs that progressed for five months. Magnetic resonance imaging (MRI) showed an enlarged lumbar plexus, which continued to the cauda equina via the L3 and L4 spinal nerves. Her symptoms gradually worsened. Ten months after the onset of symptoms, the enlarged cauda equina filled the spinal canal space, and the spinal cord was swollen. A cauda equina biopsy was performed, and she was diagnosed with GCB-type DLBCL with CD10 positivity. The primary tumor was found in a mammary cyst. The autopsy study did not show apparent infiltration, except in the nervous system.

**Conclusions:**

Although there are many neurologic phenotypes of malignant lymphoma, the association between the cytological characteristics of lymphoma and the neurological phenotypes is still unclear. Several reports of CD10-positive secondary NL are available, whereas peripheral or central nervous tissue origin lymphoma cases are mostly negative for CD10. CD10 staining may be useful for distinguishing primary NL from secondary NL. NL often has a strong organotropism for peripheral nervous tissue, which makes early diagnosis challenging.

**Supplementary Information:**

The online version contains supplementary material available at 10.1186/s12883-021-02330-5.

## Background

Neurolymphomatosis (NL) is a rare manifestation of malignant lymphoma that is characterized by lymphoma cells infiltrating the peripheral nervous system (PNS) primarily or secondarily [1]. Pathological investigations are essential for the diagnosis of NL. The most common type of lymphoma is diffuse large B-cell lymphoma (DLBCL) of non-Hodgkin lymphoma (NHL) [2]. In recent years, DLBCL has been classified into germinal center B-cell (GCB) and non-GCB types according to the Hans algorithm of immunohistochemistry [3]. The two subtypes have different susceptibilities to chemotherapy agents. However, previous studies have focused on the neuropathology of NL [1, 2, 4] and only a few investigations have applied the Hans classification to assess lymphoma pathology [3, 5]. Here, we report a patient with secondary NL originating from a breast lymphoma with exclusive peripheral nerve infiltration and massive nerve enlargement, in addition to pathological analysis according to the recent immunohistochemistry classification of DLBCL [3].

## Case presentation

A 72-year-old female developed subacute numbness, severe pain and muscle weakness in the left lower extremity. Her symptoms gradually worsened, and she was admitted to our hospital five months after the onset of symptoms. She could not extend her left hip and knee joints because of pain in the left thigh to the knee. She also had muscle weakness in her left leg; manual muscle testing revealed grade 5/2 weakness (right/left) in the quadriceps, 5/2 in the hamstrings, 5/1 in the tibialis anterior and 5/3 in the gastrocnemius. The left patellar and bilateral Achilles tendon reflexes were absent. The Babinski sign was negative on both sides.

**Fig. 1:**
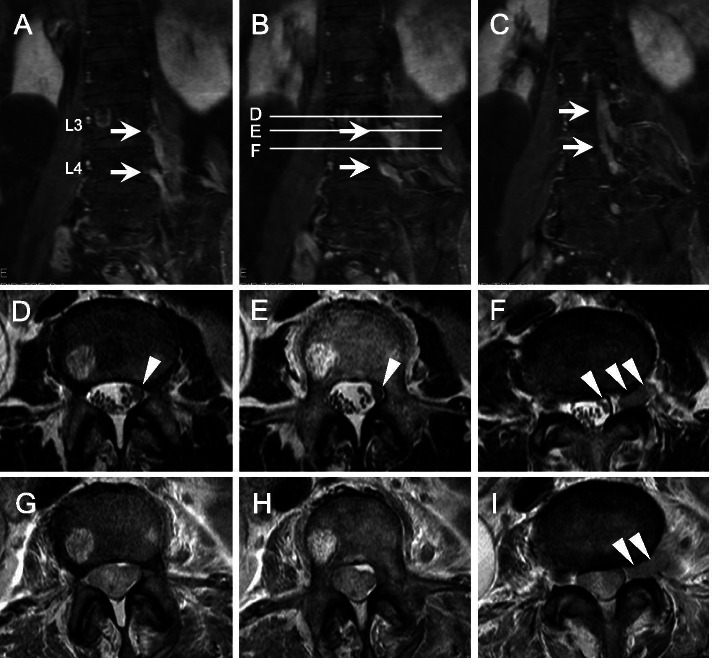
MR image of the lumbosacral spinal roots and lumbar plexus. Gadolinium-enhanced T1-weighted imaging (T1WI) MRI five months after the onset of symptoms (**A**-**F**). On the coronal view, enlarged L3 and L4 spinal nerves and lumbar plexus with marked contrast enhancement (arrows) are visible on the left side (**A**-**C**). **D**-**F** show axial T2WI slices corresponding to the lines on **B**. Enlarged nerve roots were observed (**D**-**F**, arrowheads), and the L4 nerve root filled the intervertebral foramen (**F**). In the spinal canal, the affected nerves and normal-sized nerves coexisted. **G**-**I** show T2WI slices corresponding to the levels in **D**-**F** at ten months after onset. **G**-**I** show the enlarged cauda equina that was tangled and filled the spinal canal, and **I** shows the enlarged L4 nerve root (arrowheads)

Laboratory examinations revealed that the serum soluble interleukin-2 receptor (sIL-2R) level was 445 U/mL (reference value, 220–530 U/mL), and the cerebrospinal fluid (CSF) examination showed mononuclear cell-predominant pleocytosis, with a cell count of 104/µL (< 5/µL) and an elevated protein concentration of 239 mg/dL (< 43 mg/dL). The sIL-2R level in the CSF was 590 U/mL. The CSF cytology showed only minor atypia due to inflammation. There were no malignant cells. MRI of the lumbar spine showed an enlarged lumbar plexus, which continued to the cauda equina via the L3 and L4 spinal nerves. The enlarged nerves showed marked contrast enhancement (Fig. 1A-F). Gallium scintigraphy showed no abnormal findings other than physiological accumulations.

**Fig. 2:**
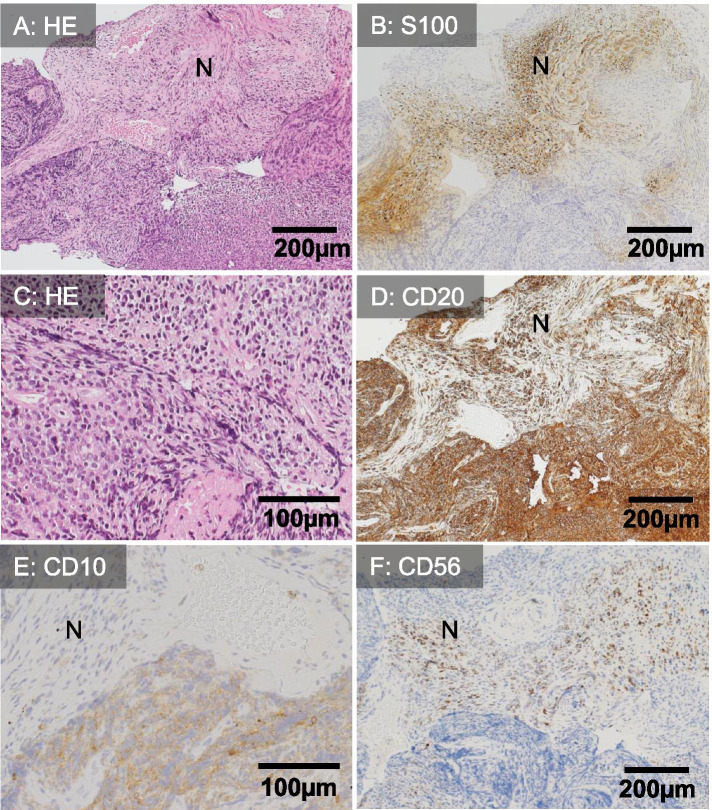
Histopathological features of the cauda equina biopsy specimen. Medium-to-large lymphoid cells filled the subdural space to the subarachnoid interstitial tissue and infiltrated peripheral nerve bundles (**A**-**C**). Nerve fibers (labeled “N”) were identified by S100 staining (polyclonal, ready to use (RTU), Leica biosystems, Wetzlar, Germany) (**C**). Immunostaining of atypical lymphoid cells showed a positive reaction for CD20 (clone L26, RTU, Leica) and CD10 (clone 56C6, RTU, Leica) and a negative reaction for CD56 (clone CD564, RTU, Leica), consistent with a diagnosis of diffuse large B-cell lymphoma of the germinal center B-cell subtype (**D**-**F**)

We strongly suspected malignancy because of localized nerve root lesions with strong enhancement on MRI and severe pain. The patient refused biopsy of the enlarged nerve because of the risk of neurological sequelae. Ten months after the onset of symptoms, she was readmitted to our hospital because of paraplegia and sensory loss in both lower extremities with sphincter impairment. In the follow-up MR study, the enlarged cauda equina was tangled and filled the spinal canal space (Fig. 1G-I), and the spinal cord was swollen with intracord T2 high signal intensity, which continued from the lumbar to the cervical level. At that time, a CSF examination was impossible because of the decreased spinal canal space. She underwent a cauda equina biopsy, and the intraoperative findings showed that the cauda equina was surrounded by a soft solid tumor. Histopathological analysis revealed that medium-to-large B-lymphoid cells filled the subdural space and subarachnoid interstitial tissue and infiltrated peripheral nerve bundles (Fig. 2A-C). Immunostaining showed that the atypical lymphoid cells were positive for CD20, CD10, MUM1, bcl2 and bcl6 but negative for CD5, CD23, CD43, CD56 and CD138 (Fig. 2D-F, Supplementary Figure 1). Ki-67 was positive in almost all viable neoplastic cell nuclei (Supplementary Figure 1). The patient was diagnosed with DLBCL of the GCB subtype according to the recent pathological classification by the WHO and Hans et al. [3, 6] The left mammary cyst, which had been identified 20 years prior, was biopsied, and similar pathologic features were confirmed. The patient underwent two courses of high-dose methotrexate therapy. However, unfortunately, she succumbed to bacterial pneumonia 13 months after the onset of symptoms.

## Discussion and conclusions

Malignant lymphoma occasionally causes various types of peripheral neuropathies, and NL is the most frequent type [2]. Most NL cases are related to NHL, especially DLBCL, which is the most common type [2]. NL is frequently accompanied by spontaneous pain, and 40% of NL cases show MRI abnormalities such as nerve enlargement or enhancement [1]. The duration from the development of neuropathy to diagnosis has been reported to be 41.3 ± 37.7 months [2]. One study showed that the median overall survival time was approximately 10 months [4]. It has been suggested that the prognosis of NL is poor because early diagnosis is difficult, and many cases are intractable to treatments. Histopathological evaluations are essential for the early diagnosis of NL and improved outcomes.

Distinguishing primary NL from secondary NL is important for therapeutic strategies. In our patient, lymphoma cells indicated CD10-positive GCB-type DLBCL. In recent years, DLBCL has been classified into GCB-type and non-GCB-type based on the Hans algorithm of immunohistochemistry [3]. Both subtypes have different susceptibilities to chemotherapy agents. In general, the GCB type has a better prognosis; however, our patient had GCB-type DLBCL [6]. More than 90% of primary central nervous system (CNS) lymphomas are reported to be non-GCB-type and tend to have a lower positivity rate for CD10 than systemic DLBCL [7, 8]. In secondary NL, there are a few reports of CD10-positive GCB-type DLBCL [9, 10] and follicular lymphoma [11], whereas CD10 positivity in definite primary NL has not been reported. Therefore, we speculated that the patient’s lymphoma was unlikely to originate from the CNS or PNS. However, because the pathological infiltrations in biopsied nerve samples are small in primary NL, the cytological characteristics of primary NL are not fully understood.

Our patient showed selective peripheral nerve involvement during the course of illness, even though her primary tumor was speculated to originate from the mammary gland. Malignant lymphoma occasionally shows strong organotropism for the PNS. The tumor microenvironment, including tumor cells, multiple stromal cells or antitumor inflammatory cells, is considered to be associated with cancer pathogenesis and progression [12]. However, the microenvironment that generates organotropism for the nervous system remains unclear. CD56 is known as a neural cell adhesion molecule [13]. Peripheral T-cell lymphoma with CD56 expression was reported to show various extranodal involvement, including the CNS [13]. In contrast, there have been no reports of NL caused by CD56-positive B-cell lymphoma. CD56 is unlikely to be the factor responsible for organotropism for the PNS. CD44, which is known as a homing cellular adhesion molecule, is also expressed on some NHL cells. In the PNS, CD44 is expressed on Schwann cells and perineurial cells in spinal nerve roots and plays an important role in cell-to-cell and cell-to-matrix adhesions [14]. These adhesion mechanisms possibly lead to PNS infiltration [14]. In our patient, CD56 staining was also negative, and CD44 staining was not performed.

NL must be considered for the differential diagnosis of hypertrophic peripheral neuropathy. NL often has strong organotropism for nervous tissue, which makes the diagnosis difficult. Early histopathological diagnosis is important. However, the cytological characteristics of NL remain unclear. We reveal the possible value of CD10 staining for the diagnosis of NL and predicting tumor extension and prognosis.

## Supplementary Information

**Additional file 1: Supplementary Figure S1**. Additional immunostaining of the cauda equina biopsy specimen. Inaddition to CD10 and CD20 immunostaining, lymphoma cells were positivefor MUM1 (clone MUM1p, 1:100, Dako, Glostrup, Denmark), bcl6 (clone LN22, readyto use (RTU), Leica biosystems, Wetzlar, Germany) and bcl2 (clone bcl-2/100/D5,RTU, Leica) (A-C) but negative for CD5 (clone 4C7, RTU, Leica), CD43(clone DF-T1, 1:1, Dako), CD23 (clone 1B12, 1:1, Nichirei, Tokyo, Japan) andCD138 (clone MI15, RTU, Leica) (D-G). Ki-67(clone MIB1, 1:100, Dako, Glostrup, Denmark) was positive in almost all viableneoplastic cell nuclei (H). EBER1 in situ hybridization (RTU, Leica) was negative (I). Nerve fibers are labeled as “N”.

## Data Availability

Further clinical data are available from the corresponding author upon reasonable request.

## Abbreviations

CNS: Central nervous systemCSF: cerebrospinal fluid
DLBCL: Diffuse large B-cell lymphoma
GCB: Germinal center B-cell
MRI: Magnetic resonance imaging
NHL: Non-Hodgkin lymphoma
NL: Neurolymphomatosis
PNS: Peripheral nervous system
sIL-2R: Soluble interleukin-2 receptor

## References

[1] Baehring JM, Damek D, Martin EC, Betensky RA, Hochberget FH (2003). Neurolymphomatosis Neuro-Oncology.

[2] Tomita M, Koike H, Kawagashira Y, Iijima M, Adachi H, Taguchi J (2013). Clinicopathological features of neuropathy associated with lymphoma. Brain.

[3] Hans CP, Weisenburger DD, Greiner TC, Gascoyne RD, Delabie J, Ott G (2004). Confirmation of the molecular classification of diffuse large B-cell lymphoma by immunohistochemistry using a tissue microarray. Blood.

[4] Kamiya-Matsuoka C, Shroff S, Gildersleeve K, Hormozdi B, Manning JT, Woodmana KH (2014). Neurolymphomatosis: a case series of clinical manifestations, treatments, and outcomes. J Neurol Sci.

[5] Khurana A, Novo M, Nowakowski GS, Ristow KM, Spinner RJ, Hunt CH (2021). Clinical manifestations of, diagnostic approach to, and treatment of neurolymphomatosis in the rituximab era. Blood Adv.

[6] Swerdlow SH, Campo E, Pileri SA, Harris NL, Stein H, Siebert R (2016). The 2016 revision of the World Health Organization classification of lymphoid neoplasms. Blood.

[7] Preusser M, Woehrer A, Koperek O, Rottenfusser A, Dieckmann K, Gatterbauer B (2010). Primary central nervous system lymphoma: a clinicopathological study of 75 cases. Pathology.

[8] Camilleri-Broët S, Crinière E, Broët P, Delwail V, Mokhtari K, Moreau A (2006). A uniform activated B-cell–like immunophenotype might explain the poor prognosis of primary central nervous system lymphomas: analysis of 83 cases. Blood.

[9] Ono Y, Kazuma Y, Ochi Y, Matsuoka R, Imai Y, Ishikawa T (2017). Two cases of neurolymphomatosis with fatal bilateral vocal cord paralysis that were diagnosed with 18F-fluorodeoxyglucose positron emission tomography (FDG PET)/CT. Intern Med.

[10] Asanome A, Kano K, Takahashi K, Saito T, Sawada J, Katayama T (2018). A case of neurolymphomatosis that was diagnosed by acoustic nerve biopsy. Clin Neurol.

[11] Umeda M, Kondo T, Nishikori M, Kitano T, Hishizawa M, Kadowaki N (2016). A case of neurolymphomatosis caused by follicular lymphoma successfully treated with bendamustine. Clin Case Rep.

[12] Hanahan D, Weinberg RA (2011). Hallmarks of cancer: the next generation. Cell.

[13] Kern WF, Spier CM, Hanneman EH, Miller TP, Matzner M, Grogan TM (1992). Neural cell adhesion molecule-positive peripheral T-cell lymphoma: a rare variant with a propensity for unusual sites of involvement. Blood.

[14] Baltuch GH, Tribolet N, Van Meir EG (1995). Expression of the CD44 adhesion molecule in tumours of the central and peripheral nervous system. J Neurooncol.

