# Supplementary figures and images for: A case report of secondary neurolymphomatosis showing selective nerve infiltration and massive lumbar plexus enlargement
Source: BMC Neurol. 2021 Jul 27;21:296. doi: 10.1186/s12883-021-02330-5 (PMC8314556; doi:10.1186/s12883-021-02330-5)

## Slide 1
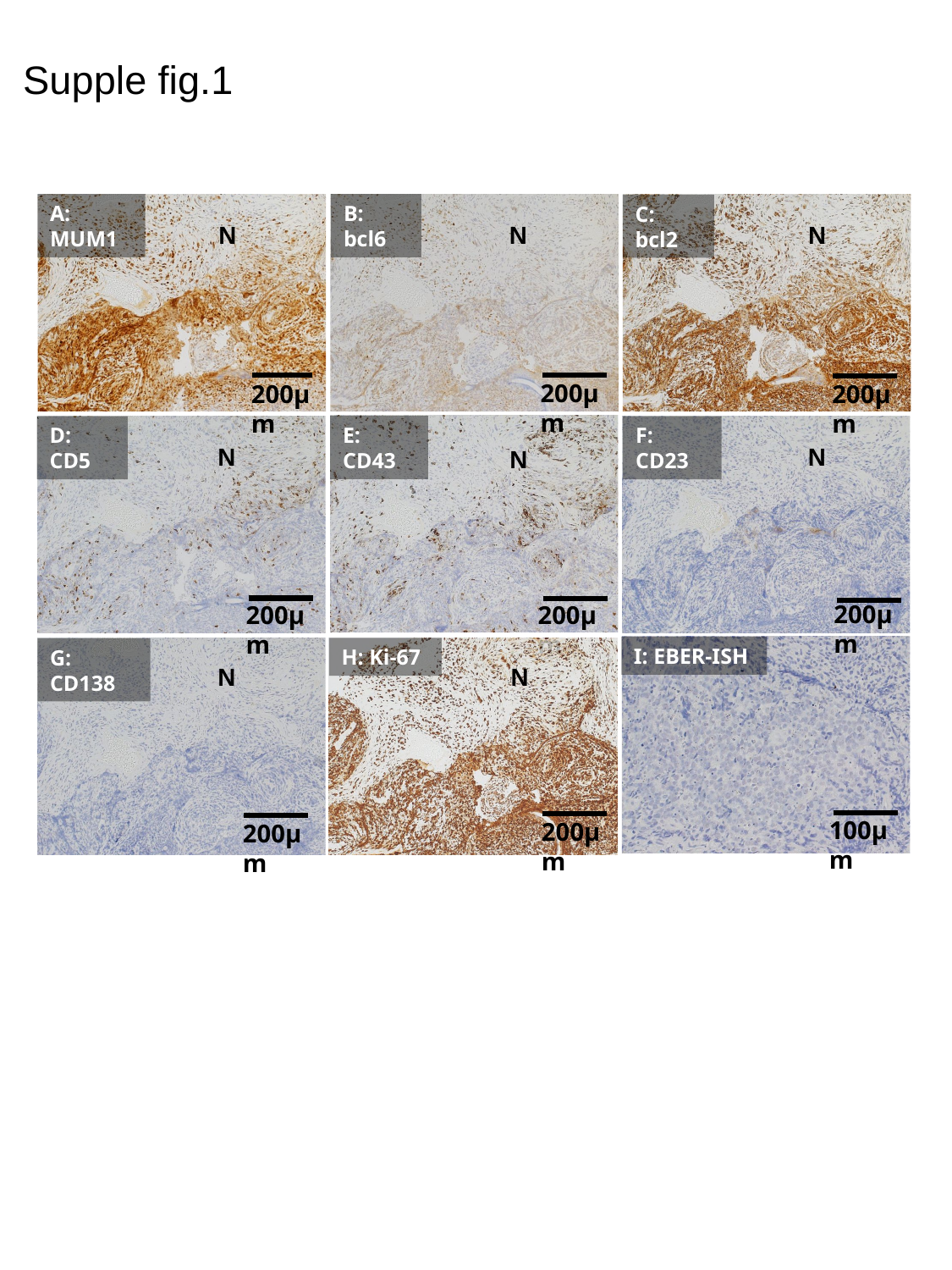

Supple fig.1
A: MUM1
B: bcl6
C: bcl2
N
N
N
200µm
200µm
200µm
E: CD43
D: CD5
F: CD23
N
N
N
200µm
200µm
200µm
I: EBER-ISH
H: Ki-67
G: CD138
N
N
100µm
200µm
200µm

Supplement: Supplementary file 1 — Additional file 1: Supplementary Figure S1. Additional immunostaining of the cauda equina biopsy specimen. Inaddition to CD10 and CD20 immunostaining, lymphoma cells were positivefor MUM1 (clone MUM1p, 1:100, Dako, Glostrup, Denmark), bcl6 (clone LN22, readyto use (RTU), Leica biosystems, Wetzlar, Germany) and bcl2 (clone bcl-2/100/D5,RTU, Leica) (A-C) but negative for CD5 (clone 4C7, RTU, Leica), CD43(clone DF-T1, 1:1, Dako), CD23 (clone 1B12, 1:1, Nichirei, Tokyo, Japan) andCD138 (clone MI15, RTU, Leica) (D-G). Ki-67(clone MIB1, 1:100, Dako, Glostrup, Denmark) was positive in almost all viableneoplastic cell nuclei (H). EBER1 in situ hybridization (RTU, Leica) was negative (I). Nerve fibers are labeled as “N”. [file 12883_2021_2330_MOESM1_ESM.pptx]
